# Textilome abdominal: à propos d'un cas

**DOI:** 10.4314/pamj.v9i1.71185

**Published:** 2011-05-26

**Authors:** Issam Serghini, Abdelghani El Fikri, Jaafar Salim Lalaoui, Mohamed Zoubir, Mohammed Boui, Mohamed Boughanem

**Affiliations:** 1 Hôpital militaire Avicenne, Service d'Anesthésie réanimation, Marrakech, Maroc; 2 Hôpital militaire Avicenne, Service de Radiologie, Marrakech, Maroc; 3 Hôpital militaire Avicenne, Service de Dermatologie, Marrakech, Maroc

**Keywords:** Textilome, chirurgie abdominale, compresses, Maroc

## Abstract

Le textilome est une complication postopératoire très rare mais bien connue. Il peut s'agir d'un corps étranger composé de compresse(s) ou champ(s) chirurgicaux laissés au niveau d'un foyer opératoire. La découverte du textilome abdominale est généralement tardive. L'anamnèse est donc essentielle pour diagnostic vu que la clinique n'est pas concluante. La clinique associe des troubles chroniques du transit à des syndromes sub-occlusifs, le cliché d'abdomen sans préparation est peu contributif. L’échographie est fiable. La tomodensitométrie permet un diagnostic topographique précis. Certaines équipes proposent des explorations par IRM. Nous rapportons un cas de textilome intra abdominale, chez une patiente opérée 6 mois auparavant d'un fibrome utérin.

## Introduction

Le textilome, également appelé gossybipoma, est une complication postopératoire très rare mais bien connue. Gossypiboma est un terme dérivé de gossypium signifiant coton en Latin et boma signifiant lieu de cachette en Swahili. Il est utilisé pour décrire un corps étranger composé de compresse(s) ou champ(s) chirurgicaux laissés au niveau d'un foyer opératoire [1, 2). C'est une complication peu fréquente de la chirurgie abdominale et pelvienne, difficile à estimer [2]. Nous rapportons un cas de textilome intra abdominale, chez une patiente opérée 6 mois auparavant d'un fibrome utérin.

## Patient et observation

Il s'agit d'une patiente de 41 ans, mariée et mère de 3 enfants, originaire et habitant Marrakech. Ses antécédents se résument en un vitiligo depuis l’âge de 30 ans et une myomectomie il y 4 mois dans un hôpital de la santé publique (cicatrice pelvienne). Elle consulte aux urgences pour des douleurs péri ombilicales modérées évoluant par crises depuis plusieurs semaines associé à des épisodes de diarrhées et de constipation sans vomissements ni d'amaigrissement et ni altération de l’ état général. L'examen clinique à l'admission trouve une patiente en BEG, apyrétique avec une PA à 130/80 mmHg et une FC à 90 battements/mn. L'examen abdominal révélait une distension importante, un tympanisme majeur et une masse abdomino-pelvienne dure et douloureuse. Le toucher rectal ne ramenait pas de sang. Le reste de l'examen somatique était sans particularité. Les résultats des explorations biologiques rapportent un syndrome inflammatoire avec une CRP à 130 mg/l sans hyper leucocytose. Le reste du bilan biologique était normal.

**Figure 1:**
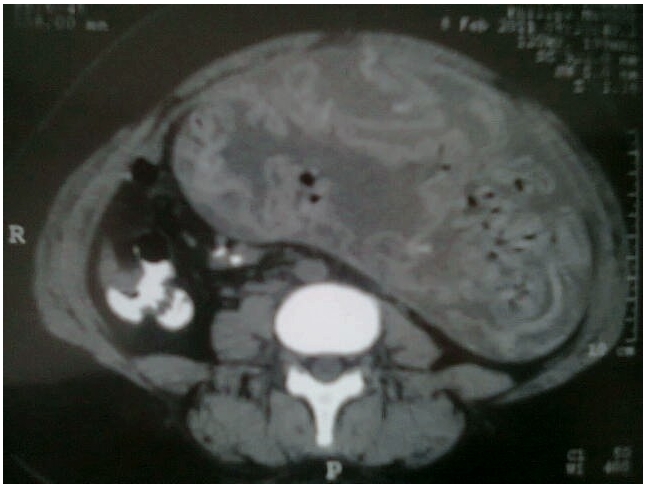
La TDM montrant une énorme masse abdomino-pelvienne mesurant 210 mm d'axe transverse sur 155 mm arrangée en accordéon, présentant entre ses plis des bulles aériques

**Figure 2:**
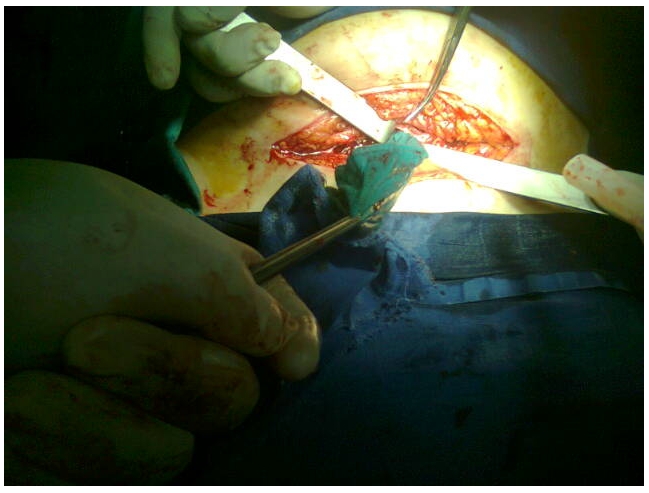
Le champ opératoire extrait de l'abdomen

Sur le plan radiologique. L'abdomen sans préparation (ASP) a mis en évidence une grande opacité sans niveaux hydroaériques. Une tomodensitométrie (TDM) est pratiquée sans et avec injection de produit de contraste. Elle met en évidence une énorme masse abdomino-pelvienne mesurant 210 mm d 'axe transverse sur 155 mm d'axe vertical, sa densité est hétérogène, composée en majorité de liquide, contenant une structure spontanément hyperdense, arrangée en accordéon, présentant entre ses plis des bulles aériques (figure 1), ces limites sont nettes, entourée d'une paroi fine, elle refoule les structures digestives sans les envahir et ne présente aucun contact avec les organes génitales. En contraste spontanée, Le foie, la rate, le pancréas et les reins sont normaux avec présence d'une autre masse latéro-utérine vraisemblablement annexielle droite. En conclusion les résultats de la TDM évoquent le diagnostic d'un textilome abdomino-pelvien. Au bloc opératoire sous anesthésie générale l'intervention chirurgicale extrait de l'abdomen un champ chirurgical moyen de 30 cm sur 30 cm (figure 2).

## Discussion

Le textilome est une lésion iatrogène développée autour d'un corps étranger textile oublié lors d'une intervention chirurgicale. Le terme plus académique de gossypiboma témoigne de la réaction inflammatoire induite par un corps textile au contact des tissus qui aboutit à la constitution d'un granulome inflammatoire [3]. La fréquence rapportée dans la littérature est de 1/1 000 à 1/10 000 [4]. La revue de la littérature (117 cas publiés de 1952 à 1993) insiste sur la prédominance des textilomes intrapéritonéaux (52%), mais d'autres sites sont concernés: gynécologiques (22%), urologiques et vasculaires (10%), osseux et rachidiens (6%), divers (10%) [5]. Le diagnostic de textilome repose sur l'examen anatomopathologique.

L'oubli de matériel reste la hantise du chirurgien lors de toute intervention et l’évolution pour le patient peut être dramatique. En effet, dans la revue de littérature de Le Neel et al [5], l'exérèse du textilome aboutit certes à la guérison sans complication chez 70 patients (59,8%),mais les complications ont aggravé l’évolution de 25 malades (21,3%),et 22 patients sont décédés (18,9%).Vingt et un des 22 décès sont à imputer aux textilomes abdominaux et concernent des textilomes symptomatiques reconnus tardivement, ayant nécessité des gestes plus agressifs (résection intestinale et/ou colique) avec un pourcentage non négligeable de complications sévères, en particulier septique.

Sur le plan physiopathologique, les fibres de textile provoquent dès la 24e heure une réaction inflammatoire avec exsudation suivi par la formation d'un tissu de granulation (8^e^ jour), enfin la fibrose s'organise à partir du 13^e^ jour. Cette évolution explique, en absence d'infection, les possibilités d'enkystement voire des calcifications avec une tolérance parfois longue [3].

La découverte du textilome abdominale est généralement tardive [6]. L'anamnèse est donc essentielle dans l’élaboration du diagnostic. La clinique manque de spécificité. Elle associe des troubles chroniques du transit à des syndromes sub-occlusifs à répétition, comme dans notre observation. Ces troubles pourraient être liés à des phénomènes de digestion du corps étranger ou à une désinvagination spontanée. Radiologiquement, le cliché d'abdomen sans préparation est peu contributif, comme souvent dans les syndromes pré-occlusifs. L’échographie est fiable et elle montre de multiples bulles d'air extra-digestives ou intra- lésionnelles sans notion d'infection. Ces bulles correspondent à l'air enchâssé dans les mailles d'une compresse en coton ou d'un champ. Les calcifications sont souvent inexistantes [7].

La tomodensitométrie permet un diagnostic topographique pré-opératoire précis. Elle réalise en même temps une exploration complète de la cavité abdominale à la recherche de complications (fistules, pneumopéritoine, abcès). Certaines équipes proposent des explorations par IRM [6, 8). En effet le textilome abdominale peut mimer une tumeur conjonctive et l'intestin grêle est une localisation fréquente des formes primitives de lymphome. Chez notre patiente, le diagnostic de tuberculose péritonéale devait être discuté. Toutefois, la lésion de notre patient n’était accompagnée ni d'ascite, ni d'adénomégalie péri-pancréatique et du petit épiploon, ni de nécrose centrale. Le textilome peut être confondu avec un adénocarcinome colique [9]. Le contexte clinique et l'altération de l’état général présent en cas de tumeur chez un patient jeune aident à redresser le diagnostic. D'autre part, la corrélation entre les explorations échographiques, tomodensitométriques et macroscopiques permet de mieux comprendre et analyser les images.

Le comptage des compresses et des champs par le chirurgien en début et fin d'intervention reste un moyen efficace mais encore insuffisant. Aux Etats unis, l'utilisation de compresses marquées radio-opaques dès 1940 selon les recommandations de Cr Ossen, a contribué de façon significative à limiter ce type d'incident [5].

## Conclusion

Malgré les avancées actuelles, la prudence reste de mise concernant les compresses chirurgicales ou champs opératoires sur les sites précédemment opérés, qui peuvent être responsables de granulomes pseudo-tumoraux, causant des dégâts tissulaires importants autour du corps étranger accidentellement laissé en place. Selon la jurisprudence et le droit médical, la découverte d'un textilome est reconnue comme une faute, entrainant la responsabilité du chirurgien.
